# N^6^-Methyladenosine Regulators and Related LncRNAs Are Potential to be Prognostic Markers for Uveal Melanoma and Indicators of Tumor Microenvironment Remodeling

**DOI:** 10.3389/fonc.2021.704543

**Published:** 2021-07-30

**Authors:** Zhicheng Liu, Shanshan Li, Shan Huang, Tao Wang, Zhicheng Liu

**Affiliations:** ^1^School of Biomedical Engineering, Capital Medical University, Beijing, China; ^2^Beijing Key Laboratory of Fundamental Research on Biomechanics in Clinical Application, Capital Medical University, Beijing, China

**Keywords:** m^6^A RNA methylation regulators, long noncoding RNAs, tumor microenvironment, immune cell infiltration, uveal melanoma

## Abstract

Uveal melanoma (UM) is one of the most common malignant intraocular tumors in adults. Few studies have investigated the effect of N^6^-methyladenosine (m^6^A) RNA methylation regulators and related long noncoding RNAs (lncRNAs) on the tumor microenvironment (TME) and survival time of patients with UM. Based on the transcriptome and clinical data from The Cancer Genome Atlas, we systematically identified m^6^A regulators. Then, we constructed an m^6^A regulators-based signature to predict the prognostic risk using univariate and LASSO Cox analyses. The signature was then validated by performing Kaplan-Meier, and receiver operating characteristic analyses. Through the correlation analysis, m^6^A regulators-related lncRNAs were identified, and they were divided into different clustering subtypes according to their expression. We further assessed differences in TME scores, the survival time of patients, and immune cell infiltration levels between different clustering subtypes. Finally, we screened out the common immune genes shared by m^6^A-related lncRNAs and determined their expression in different risk groups and clustering subtypes. For further validation, we used single-cell sequencing data from the GSE139829 dataset to explore the expression distribution of immune genes in the TME of UM. We constructed a prognostic risk signature representing an independent prognostic factor for UM using 3 m^6^A regulators. Patients in the low-risk group exhibited a more favorable prognosis and lower immune cell infiltration levels than patients in the high-risk group. Two subtypes (cluster 1/2) were identified based on m^6^A regulators-related lncRNAs. The TME scores, prognosis, and immune cell infiltration have a marked difference between cluster 1 and cluster 2. Additionally, 13 common immune genes shared by 5 lncRNAs were screened out. We found that these immune genes were differentially expressed in different risk groups and clustering subtypes and were widely distributed in 3 cell types of TME. In conclusion, our study demonstrated the important role of m^6^A regulators and related lncRNAs in TME remodeling. The signature developed using m^6^A regulators might serve as a promising parameter for the clinical prediction of UM.

## Introduction

Uveal melanoma (UM), which is the second most common type of melanoma, originates from melanocytes in the intraocular uvea (1). Currently, surgery and radiotherapy are the most effective methods to treat local tumors (2). However, the overall mortality in patients with UM is more than 50%, because it is highly susceptible to early metastasis (3, 4). Therefore, new treatments such as immunotherapy or targeted therapy (5, 6) are being developed, which requires the identification of several potential prognostic biomarkers and therapeutic targets for UM.

The most prevalent RNA modification is N^6^-methyladenosine (m^6^A), which involves methylation of the sixth N atom of adenine (7). m^6^A methylation is a dynamic process regulated by methyltransferases (writers) and demethylases (erasers), whereas binding proteins (readers) bind to m^6^A methylation sites (8, 9). Methyltransferases such as METTL3/14/16, WTAP, RBM15, and VIRMA promote m^6^A methylation (9–11). On the other hand, demethylases, which include FTO and ALKBH5, inhibit m^6^A methylation (9, 11). Binding proteins, such as YTHDC1/2, YTHDF1-3, IGF2BP1-3, and HNRNPC, bind to the m^6^A modified site to form a complex that mediates its biological function (12). These m^6^A methylases are primarily involved in mammalian development, immune response, tumorigenesis, and metastasis, and stem cell differentiation (13–16). However, the prognostic role of m^6^A methylases in UM development has not been sufficiently investigated. Besides, the involvement of m^6^A methylases in the tumor microenvironment (TME) remains to be thoroughly explored.

Long noncoding RNAs (lncRNAs) are frequently defined as RNAs that have a transcript length exceeding 200 nucleotides and do not encode proteins (17). They can regulate gene expression at epigenetic, transcriptional, and post-transcriptional levels (18). A recent study has reported that lncRNAs promote tumor development by altering the immune microenvironment (19). Increasing evidence has demonstrated the TME, which mainly consists of stromal and immune cells, plays an important role in tumor progression (20). Stromal cells may contribute to tumor angiogenesis and extracellular matrix reorganization, whereas immune cells may contribute to TME *via* dysregulation of immune-mediated responses (21). Therefore, immune cell infiltration in the TME may serve as a potential target for immunotherapy. However, the involvement of lncRNAs in immune cell infiltration in UM remains unclear.

This study aimed to systematically explore m^6^A regulators and related lncRNAs involved in the TME in UM and developed an m^6^A regulators-based signature for improving the accuracy of prognosis in patients with UM. We also established clustering subtypes based on m^6^A regulators-related lncRNAs to determine the relationships between the clustering subtypes, TME scores, prognosis, and immune cell infiltration, and further explained the mechanism of action of m^6^A regulators. Finally, we explored the expression of 13 immune genes shared by 5 lncRNAs in different risk groups and clustering subtypes.

## Materials and Methods

### Data Collection and Preparation

The RNA-sequencing transcriptome data of 80 patients with UM and corresponding clinical data were downloaded from The Cancer Genome Atlas (TCGA) data portal (https://portal.gdc.cancer.gov/). GTF files were downloaded from Ensembl (https://asia.ensembl.org) to distinguish between lncRNAs and mRNAs for subsequent analyses. The list of immune genes was downloaded from the ImmPort database (https://www.immport.org).

### Generation of TME Scores and Tumor-Infiltrating Immune Cells

The ESTIMATE algorithm in the R “estimate” package was used to calculate the TME scores of 80 UM patients. The Kaplan-Meier survival analysis was conducted to compare the difference in survival time using R “survMiner” and “survival” packages. The fraction of 22 immune cell types in each sample was estimated using CIBERSORT. The association between TME scores and tumor-infiltrating immune cells (TICs) was established using correlation analysis.

### Construction and Validation of m^6^A Regulators-Based Signature

The m^6^A regulators were identified from the published literature (9–12). The m^6^A regulators contain 8 writers (METTL3, METTL14, METTL16, WTAP, VIRMA, ZC3H13, RBM15, and RBM15B), 13 readers (YTHDC1, YTHDC2, YTHDF1, YTHDF2, YTHDF3, HNRNPC, FMR1, LRPPRC, HNRNPA2B1, IGF2BP1, IGF2BP2, IGF2BP3, and RBMX), and 2 erasers (FTO and ALKBH5). The expression data of m^6^A regulators were extracted from the mRNA expression data of TCGA. The m^6^A regulators, which were previously identified using the univariate Cox regression analysis, were further subjected to the LASSO Cox regression analysis using the “glmnet” package. The minimum 10-fold cross-validation was used to select the best penalty parameter λ. Then, the risk score of each patient was calculated using a linear combination of m^6^A regulators expression weighted by the multivariate Cox regression analysis. According to the median risk score, the samples were divided into high-risk and low-risk groups. Subsequently, the Kaplan-Meier survival analysis was performed to compare the survival difference. Receiver operating characteristic (ROC) analysis was performed to evaluate the prognostic value of the signature using the “timeROC” package.

### Generation and Validation of Clustering Subtypes From m^6^A Regulators-Related LncRNAs

We screened m^6^A regulators-related lncRNAs by Pearson’s correlation analysis. The process used the criteria of |correlation coefficient| > 0.4 and p < 0.001. The expression data of m^6^A regulators-related lncRNAs were extracted from the lncRNA expression data of TCGA. To clarify the biological characteristics of m^6^A regulators-related lncRNAs, the R “ConsensusClusterPlus” package was used to divide the samples into different clustering subtypes according to the expression of lncRNAs. Principal component analysis (PCA), Kaplan-Meier survival analysis, TME scores, and TICs profiles were performed for different clustering subtypes.

### Identification and Validation of Immune Genes Shared by 5 LncRNAs

The Pearson’s correlation analysis was performed to screen common immune genes shared by 5 lncRNAs. The Kaplan-Meier survival analysis was used to compare the survival difference between high- and low-expression of immune genes. The differential expression analysis of immune genes was performed using the R “limma” package in different risk groups and clustering subtypes. A p-value < 0.05 indicated statistical significance. At present, single-cell sequencing technology has been widely used to explore the heterogeneity of TME. To characterize immune genes expression distribution in TME of UM, we search for single-cell sequencing data of UM from Tumor Immune Single-cell Hub (TISCH) (22). The GSE139829 dataset including 59,915 cell sequencing data from 11 samples was collected to perform gene expression distribution (23).

## Results

### The Correlation Between TME Scores With the Survival of UM Patients and Immune Cell Infiltration

**Figure 1:**
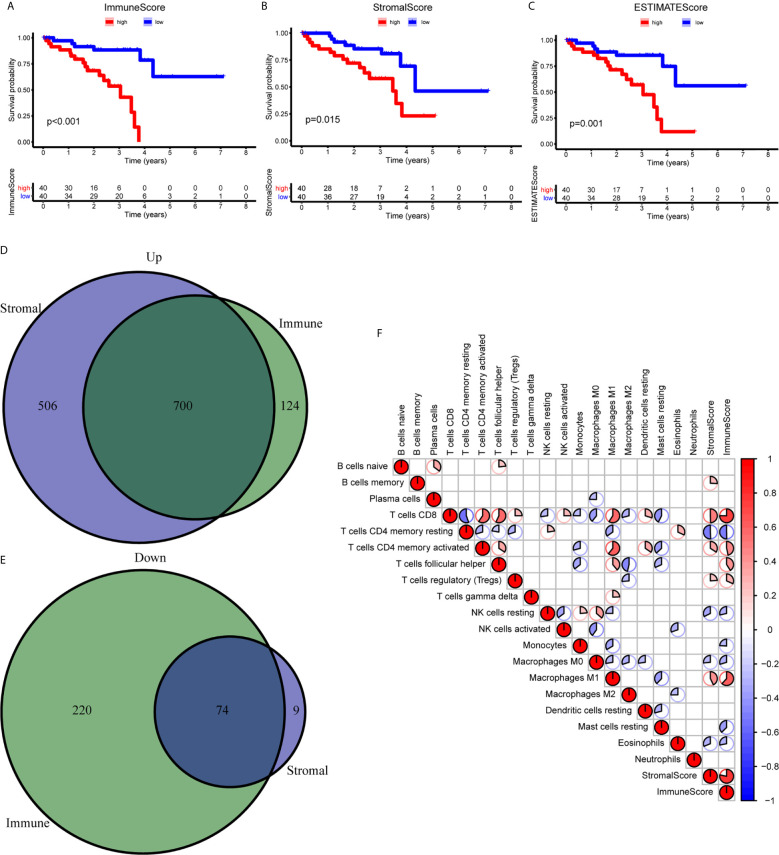
Correlation between the TME scores and survival of UM patients. **(A–C)** Kaplan-Meier survival analysis between high and low ImmuneScore **(A)**, StromalScore **(B)**, and ESTIMATEScore **(C)**. **(D, E)** The Venn diagram showed the common upregulated **(D)** and downregulated **(E)** DEGs shared by ImmuneScore and StromalScore. **(F)** The relationship between 19 immune cell types and score of immune and stromal. DEGs, differentially expressed genes.

To establish the correlation of ImmuneScore and StromalScore with the survival time, we performed the Kaplan-Meier survival analysis. A high score of immune and stromal cells signified large numbers of these cells in the TME. As shown in **Figure 1A**, the overall survival (OS) in the low ImmuneScore group was longer than that in the high ImmuneScore group. Similarly, StromalScore and ESTIMATEScore showed a negative correlation with the OS (**Figures 1B, C**). To confirm exact changes in the genetic profiles in the TME about immune and stromal cell components, variance analysis of high and low scores was performed. As shown in **Figures 1D, E**, 700 common differentially expressed genes (DEGs) were upregulated, and 74 common DEGs were downregulated. To further explore the interaction between TME scores and the 22 immune cell types, we first estimated the 22 types of TICs with abundance distribution in all the tumor samples and then calculated the correlation index between the TME scores and TICs. The results showed that ImmuneScore is correlated with CD8^+^ T cells, resting memory CD4^+^ T cells, activated memory CD4^+^ T cells, helper T cells (follicular), Tregs, resting NK cells, monocytes, M0 macrophages, M1 macrophages, resting mast cells, and eosinophils (**Figure 1F**).

### Construction and Validation of m^6^A Regulators-Based Signature

**Figure 2:**
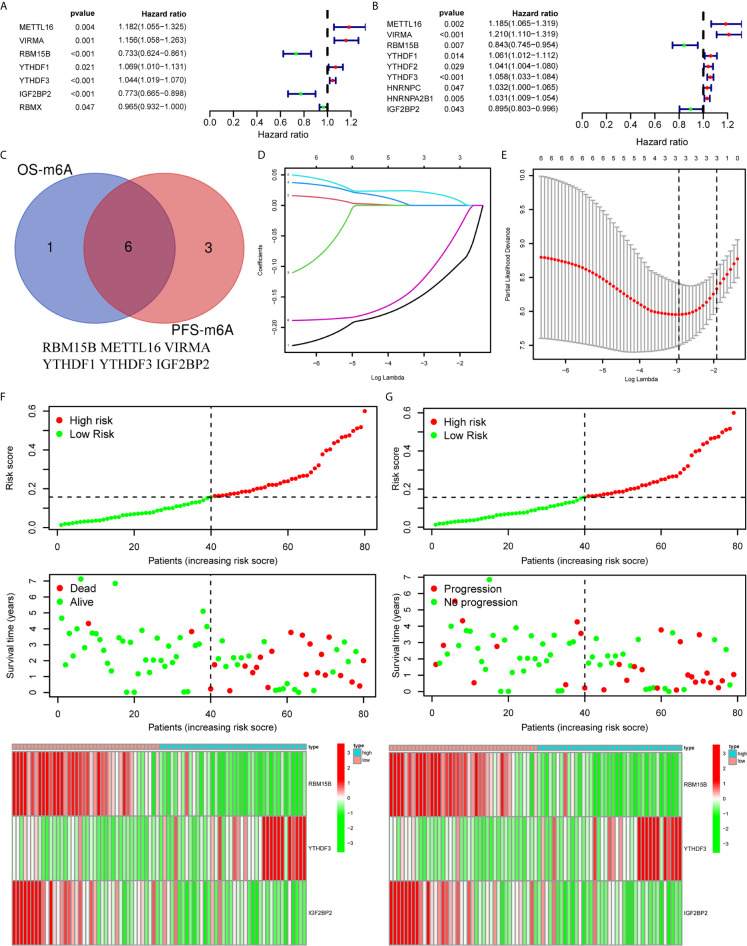
Construction of a prognostic signature based on m^6^A regulators. **(A, B)** Forest plots for the univariate Cox analysis of prognosis based on OS **(A)** and PFS **(B)**. Colored dots represent hazard ratio, and the horizontal lines across the hazard ratio represent 95% confidence interval. **(C)** The Venn plot showed 6 common m^6^A regulators based on both OS and PFS. **(D)** LASSO coefficient profiles of the 6 m^6^A regulators. **(E)** The minimum 10-fold cross-validation was used to select the best penalty parameter λ in the LASSO model. **(F, G)** The distribution of the risk score (Upper), pattern of survival time and survival status (Middle), and the heat map (Bottom) of the 3 prognostic m^6^A regulators levels based on OS **(F)** and PFS **(G)**. OS, overall survival; PFS, progression free survival.

**Figure 3:**
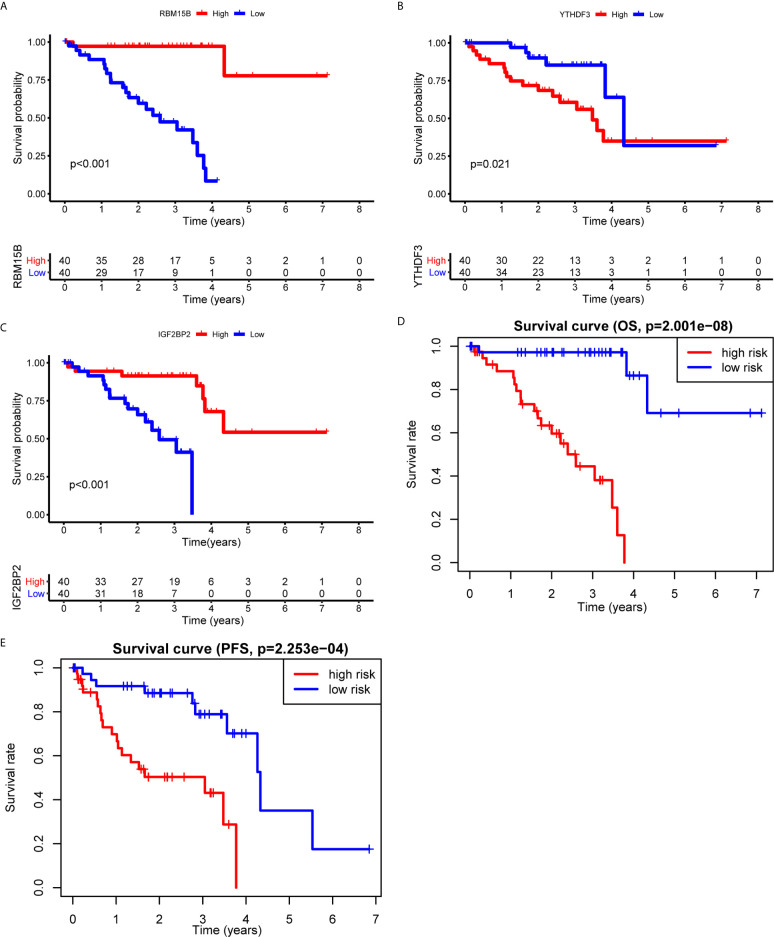
Kaplan-Meier survival analysis of the 3 m^6^A regulators and the risk signature. **(A–C)** Survival curves between the high- and low-expression of RBM15B **(A)**, YTHDF3 **(B)**, and IGF2BP2 **(C)**. **(D, E)** Survival curves of the 3 m^6^A regulators-based signature based on OS **(D)** and PFS **(E)**. OS, overall survival; PFS, progression free survival.

**Figure 4:**
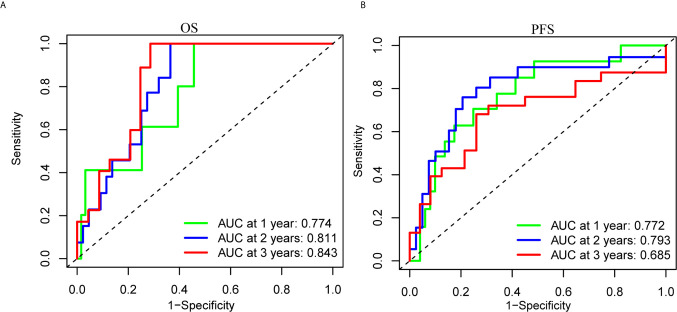
ROC analysis of the 3 m^6^A regulators-based signature. **(A, B)** The 1-, 2-, and 3-years ROC analysis of the prognostic prediction based on the 3 m^6^A regulators according to OS **(A)** and PFS **(B)**. OS, overall survival; PFS, progression free survival; AUC, area under the ROC curve.

To clarify the biological function of m^6^A regulators in the prognosis of patients with UM, we comprehensively investigated the prognostic value of m^6^A regulators based on the expression and clinical data (**Table S1**). Of these m^6^A regulators, seven exhibited a prognostic value based on OS (**Figure 2A**), while nine displayed a prognostic value based on progression-free survival (PFS) (**Figure 2B**). The Venn plot results indicated that 6 m^6^A regulators (RBM15B, IGF2BP2, YTHDF1, METTL16, VIRMA, and YTHDF3) were identified based on OS and PFS (**Figure 2C**). To avoid overfitting, we performed the LASSO Cox analysis and selected 3 of the 6 m^6^A regulators were to establish a risk signature (**Figures 2D, E**). Thus, we established a predictive model: risk score = (RBM15B * −0.14284) + (YTHDF3 * 0.02121) + (IGF2BP2 * −0.11533). The distribution of risk score (Upper), patients’ survival time (Middle), and heat map analysis (Bottom) of the 3 prognostic m^6^A regulators were shown based on the OS (**Figures 2F**) and PFS (**Figure 2G**). Results of the heat maps (**Figures 2F, G**) was survival curves (**Figures 3A–C**) suggested that YTHDF3 was likely to be a high-risk factor because it was upregulated in the high-risk group. However, the highly expressed RBM15B and IGF2BP2 in the low-risk group might be protective factors. As shown in **Figures 3D, E**, the OS and PFS of patients in the low-risk group were longer than in the high-risk group. To evaluate the prognostic accuracy of the 3 m^6^A regulators-based signature, we performed the ROC analysis based on OS and PFS. Areas under the ROC curves of 1, 2, and 3 years were 0.774, 0.811, and 0.843, respectively (**Figure 4A**). The PFS prediction of 3 m^6^A regulators-based signature was also accurate (**Figure 4B**). These results suggested that the 3 m^6^A regulators-based signature might serve as a promising parameter for prognostic prediction of UM.

### The Association Between Risk Score With Immune Cell Infiltration and TME Scores

**Figure 5:**
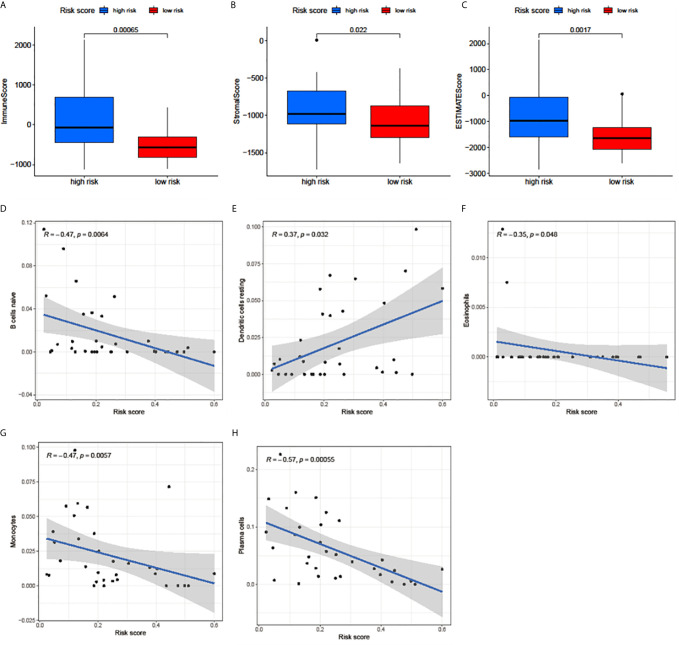
Relationships among the risk score, TME scores, and immune cell infiltration of 5 immune cell types. **(A–C)** The variance analysis of ImmuneScore **(A)**, StromalScore **(B)**, and ESTIMATEScore **(C)** in the high- and low-risk groups. **(D–H)** The correlation between risk score and naive B cells **(D)**, resting dendritic cells **(E)**, eosinophils **(F)**, monocytes **(G)**, and plasma cells **(H)**. The blue line in each plot was fitted linear model indicating the proportion tropism of immune cell along with risk score. The shade around the blue line represents the 95% confidence interval.

The correlation between the risk score and the immune cell infiltration levels was calculated to establish the association between the 3 m^6^A regulators-based signature with the TME. ImmuneScore, StromalScore, and ESTIMATEScore in the high-risk group were significantly higher than in the low-risk group (**Figures 5A–C**). The results showed that the risk score was significantly negatively correlated with the infiltration levels of naive B cells (**Figure 5D**), eosinophils (**Figure 5F**), monocytes (**Figure 5G**), and plasma cells (**Figure 5H**). Only the infiltration level of resting dendritic cells was positively correlated with the risk score (**Figure 5E**). These results indicated that the 3 m^6^A regulators were involved in the immune cell infiltration of UM.

### The Correlation Between Consensus Clustering of m^6^A Regulators-Related LncRNAs and TME Scores, Survival Time, and Immune Cell Infiltration

**Figure 6:**
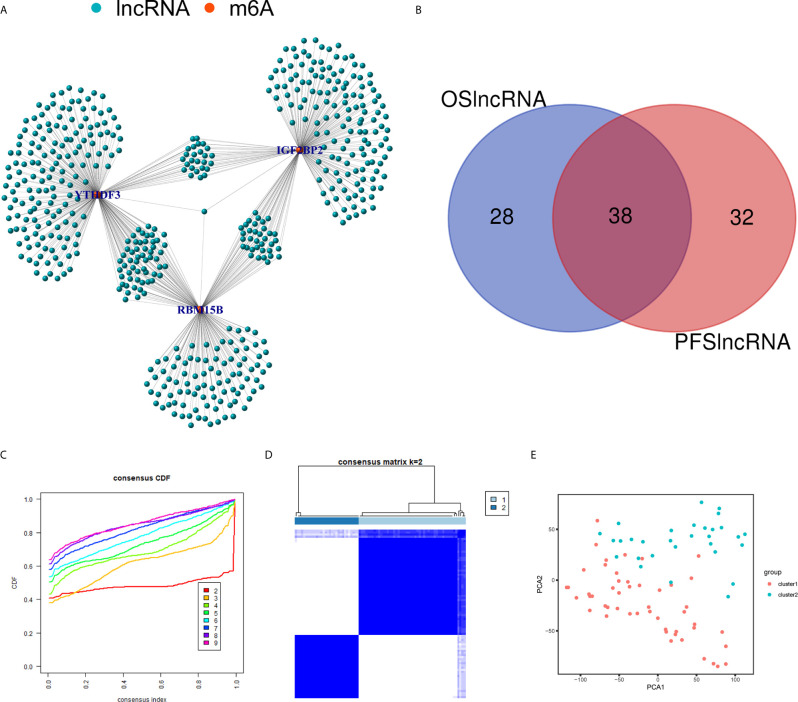
Identification of m^6^A regulators-related lncRNAs. **(A)** The network between the 3 m^6^A regulators (red dots) and lncRNAs (green dots). **(B)** The Venn plot showed 38 common lncRNAs shared by OS-related lncRNAs and PFS-related lncRNAs. **(C)** Consensus clustering cumulative distribution function (CDF) for k = 2 to 9. **(D)** Consensus clustering matrix for k = 2. **(E)** Principal component analysis (PCA) of 2 subtypes based on 38 lncRNAs for each sample. OS, overall survival; PFS, progression free survival.

Using the correlation analysis, we identified 514 lncRNAs based on RNA-Seq data and constructed a network between m^6^A regulators and lncRNAs (**Figure 6A**). A total of 66 of the 514 lncRNAs had a prognostic value based on the OS (**Figure S1A**), while 70 of the 514 lncRNAs had a prognostic value based on the PFS (**Figure S1B**). The Venn plot results showed that 38 lncRNAs were common according to OS and PFS (**Figure 6B**). Based on the similarity identified by consensus clustering using the ‘ConsensusClusterPlus’ package, we found that k = 2 was the optimal clustering stability value (**Figures 6C, D**). The 80 UM samples were well-differentiated into two subtypes according to the expression of 38 lncRNAs (**Figure 6E**).

**Figure 7:**
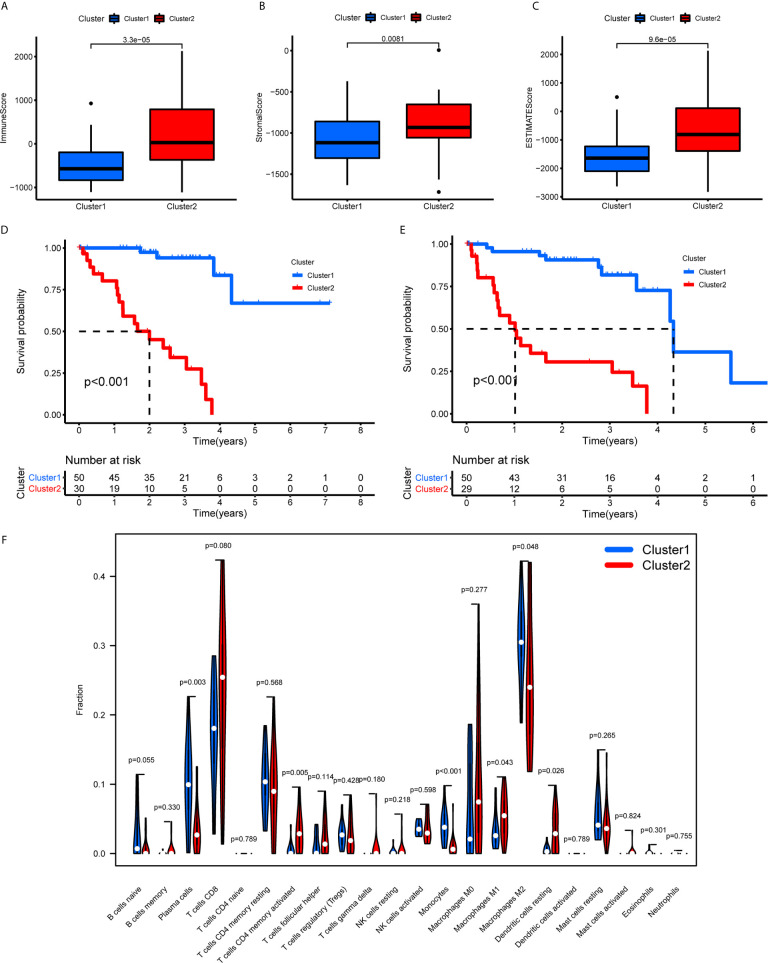
TME scores, survival analysis for UM, and TICs in cluster 1/2 subtypes constructed by 38 m^6^A regulators-related lncRNAs. **(A–C)** The variance analysis of ImmuneScore **(A)**, StromalScore **(B)**, and ESTIMATEScore **(C)** in cluster 1/2 subtypes. **(D, E)** Kaplan-Meier survival analysis of OS **(D)** and PFS **(E)** for patients with UM in cluster 1/2 subtypes. **(F)** The violin plot showed the fraction differentiation of 22 kinds of immune cells in cluster 1/2 subtypes. TICs, tumor-infiltrating immune cells; OS, overall survival; PFS, progression free survival.

The TME scores of cluster 1 were lower than those of cluster 2 (**Figures 7A–C**), while the OS (**Figure 7D**) and PFS (**Figure 7E**) of cluster 1 were notably longer than those of cluster 2. Subsequently, the 22 immune cell levels for the two subtypes were calculated. The results showed that cluster 1 had higher immune infiltration levels of plasma cells, and monocytes while there were higher immune cell infiltration levels of activated memory CD4^+^ T cells, follicular helper T cells, M1 macrophages, and resting dendritic cells (**Figure 7F**).

### Identification and Validation of Immune Genes Shared by 5 LncRNAs

**Figure 8:**
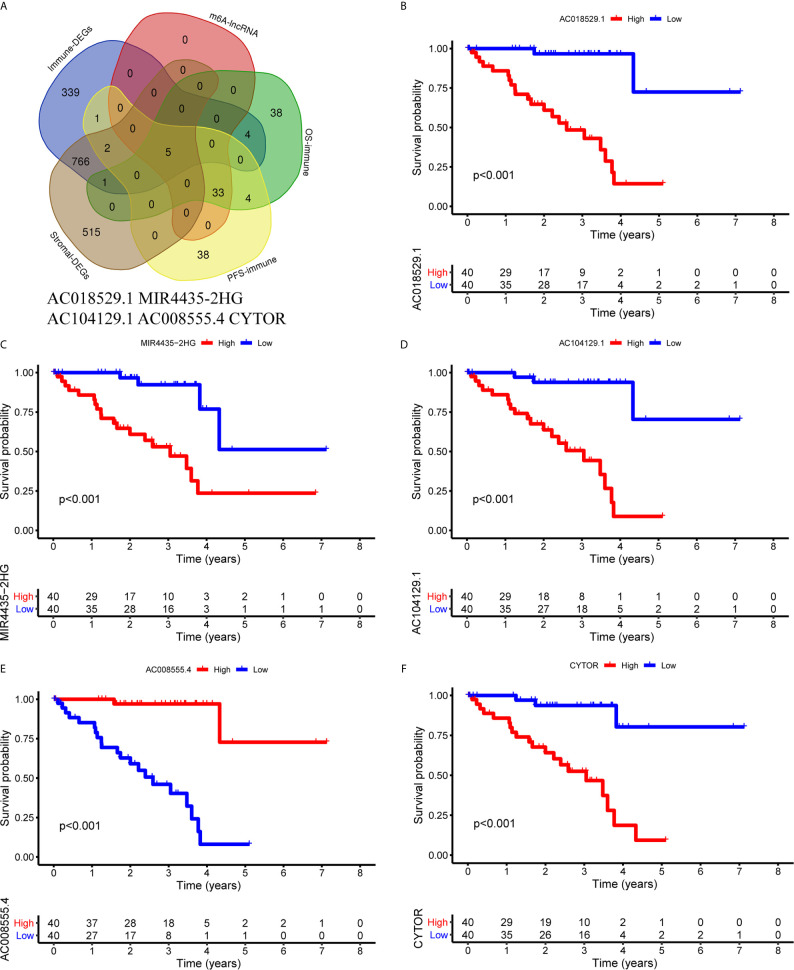
Identification of common lncRNAs from 5 lists. **(A)** The Venn plot showed 5 common lncRNAs in 5 lists. They were ImmuneScore (Immune-DEGs) and StromalScore (Stromal-DEGs), m^6^A regulators-related lncRNAs (m^6^A-lncRNAs), and immune gene-specific lncRNAs based on OS (OS-immune) and PFS (PFS-immune). **(B–F)** Survival curves based on the high or low expression of AC018529.1 **(B)**, MIR4435-2HG **(C)**, AC104129.1 **(D)**, AC008555.4 **(E)**, and CYTOR **(F)**. DEGs, differentially expressed genes.

**Figure 9:**
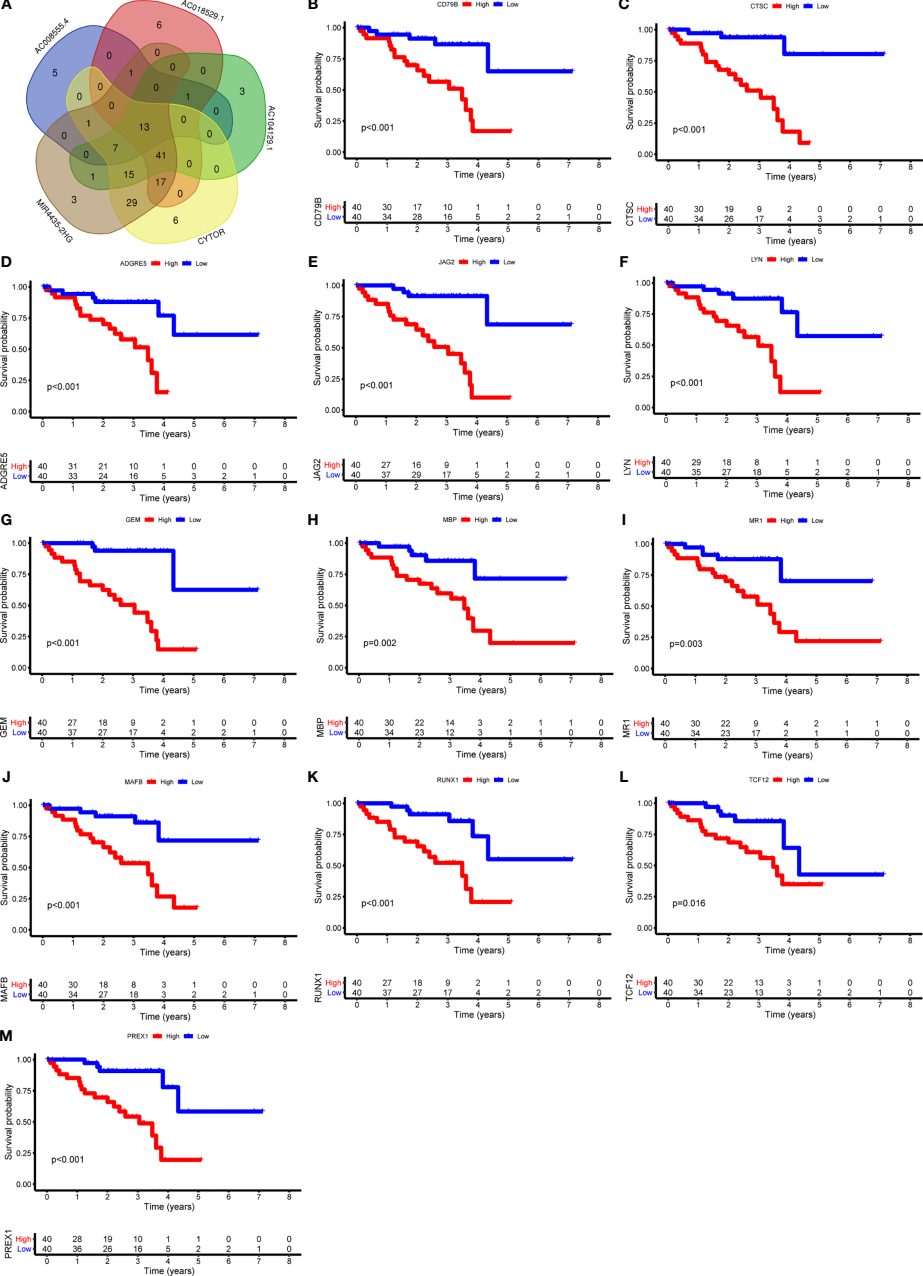
Identification of immune genes targeted by lncRNAs. **(A)** 13 immune genes shared by 5 lncRNAs were identified. (B-M) Survival curves based on the high or low expression of CD79B **(B)**, CTSC **(C)**, ADGRE5 **(D)**, JAG2 **(E)**, LYN **(F)**, GEM **(G)**, MBP **(H)**, MR1 **(I)**, MAFB **(J)**, RUNX1 **(K)**, TCF12 **(L)**, and PREX1 **(M)**. High expression of these immune genes was associated with a poor prognosis.

**Figure 10:**
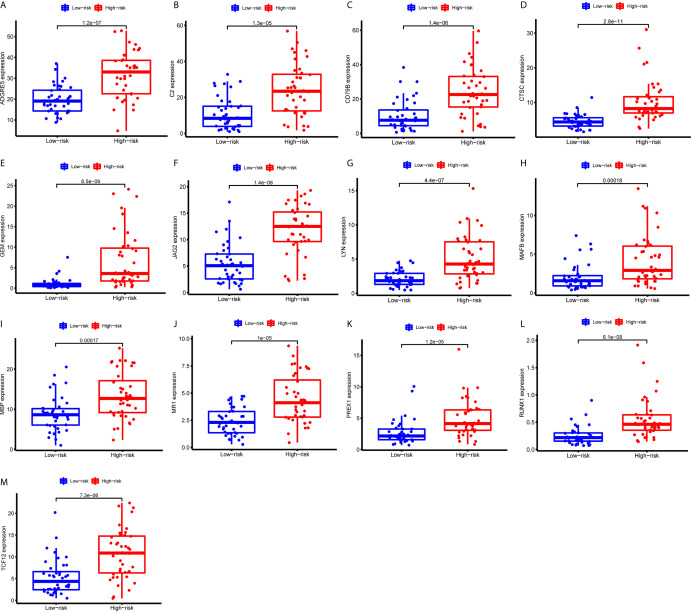
Expression of the 13 immune genes in the high- or low-risk groups based on 3 m^6^A regulators. (A-M) The differential expression analysis of ADGRE5 **(A)**, C2 **(B)**, CD79B **(C)**, CTSC **(D)**, GEM **(E)**, JAG2 **(F)**, LYN **(G)**, MAFB **(H)**, MBP **(I)**, MR1 **(J)**, PREX1 **(K)**, RUNX1 **(L)**, and TCF12 **(M)** between the high- and low-risk groups. The expression levels of 13 immune genes were all upregulated in the high-risk group than those in the low-risk group.

**Figure 11:**
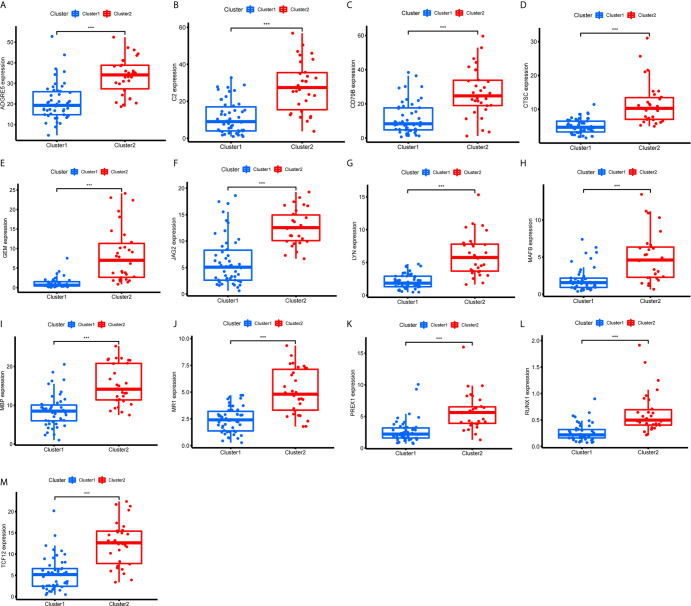
Expression of the 13 immune genes in cluster 1/2 based on m^6^A-related lncRNAs. **(A–M)** The differential expression analysis of ADGRE5 **(A)**, C2 **(B)**, CD79B **(C)**, CTSC **(D)**, GEM **(E)**, JAG2 **(F)**, LYN **(G)**, MAFB **(H)**, MBP **(I)**, MR1 **(J)**, PREX1 **(K)**, RUNX1 **(L)**, and TCF12 **(M)** between cluster 1 and cluster 2. The expression levels of 13 immune genes in cluster 2 were significantly increased compared with those in cluster 1. ^***^P < 0.001.

**Figure 12:**
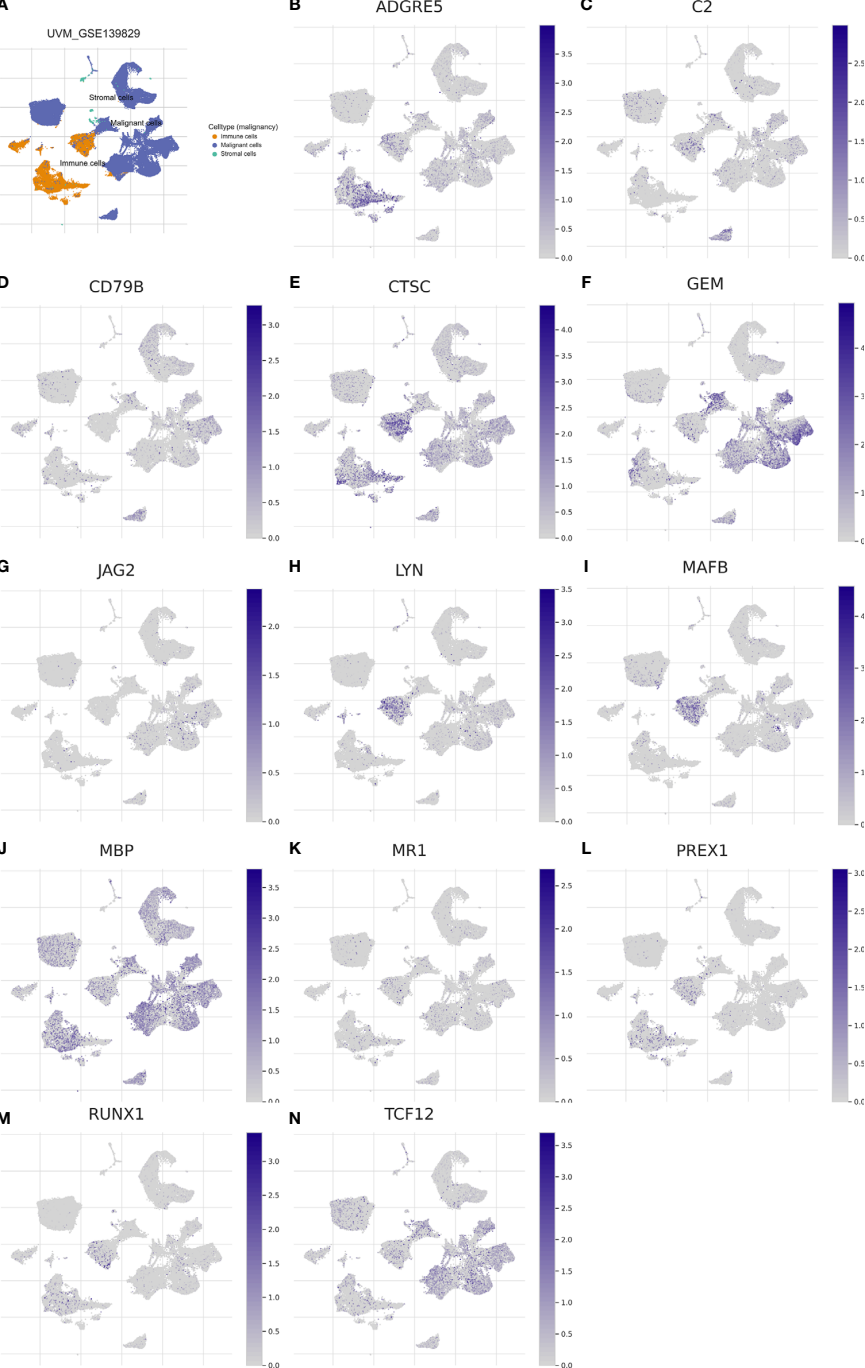
Expression distribution of the 13 immune genes in TME of UM. **(A)** The overview tab of 3 cell types from the GSE139829 dataset. The colored shapes (left) showed the cell distribution and the cell type annotations were displayed on the right side. orange dot: immune cells; blue dot: malignant cells; green dot: stromal cells. **(B–N)** The expression distribution of ADGRE5 **(B)**, C2 **(C)**, CD79B **(D)**, CTSC **(E)**, GEM **(F)**, JAG2 **(G)**, LYN **(H)**, MAFB **(I)**, MBP **(J)**, MR1 **(K)**, PREX1 **(L)**, RUNX1 **(M)**, and TCF12 **(N)** in 3 cell types. The colored dots indicate the distribution of immune genes in the corresponding cell type.

First, we identified the common lncRNAs from 5 lists that were DEGs in ImmuneScore (Immune-DEGs) and StromalScore (Stromal-DEGs), m^6^A regulators-related lncRNAs (m^6^A-lncRNAs), and immune gene-specific lncRNAs based on OS (OS-immune) and PFS (PFS-immune). A total of 5 lncRNAs, namely AC008555.4, AC018529.1, AC104129.1, CYTOR, and MIR4435-2HG, were found to be common across the 5 lists (**Figure 8A**). High expression of AC018529.1 (**Figure 8B**), MIR4435-2HG (**Figure 8C**), AC104129.1 (**Figure 8D**), and CYTOR (**Figure 8F**) was related to the poor prognosis of patients, while high expression of AC008555.4 was associated with good prognosis (**Figure 8E**). Next, 13 immune genes having 5 common lncRNAs were screened using the correlation analysis (**Figure 9A**). The 13 immune genes were ADGRE5, C2, CD79B, CTSC, GEM, JAG2, LYN, MAFB, MBP, MR1, PREX1, RUNX1, and TCF12. Except for C2, the other 12 immune genes were associated with a poor prognosis (**Figures 9B–M**). To further verify whether the immune genes were differentially expressed in different groups based on m^6^A regulators and lncRNAs, we extracted the expression data from RNA-Seq data and performed the differential expression analysis. The expression levels of 13 immune genes in the high-risk group were upregulated than those in the low-risk group (**Figure 10**). Similarly, the 13 immune genes levels in cluster 2 were upregulated compared with cluster 1 (**Figure 11**). For further validation, we used TISCH to depict the expression distribution of 13 immune genes in the TME of UM. The overall distribution of the 3 cell types in the GSE139829 dataset was shown in **Figure 12A**. Through analysis, we found that the expression distribution of ADGRE5 (**Figure 12B**), CTSC (**Figure 12E**), LYN (**Figure 12H**), MAFB (**Figure 12I**), PREX1 (**Figure 12L**), and RUNX1 (**Figure 12M**) were abundant in immune cells. CD79B (**Figure 12D**), GEM (**Figure 12F**), and JAG2 (**Figure 12G**) expression distribution were concentrated in malignant cells. The gene expression of C2 (**Figure 12C**), MBP (**Figure 12J**), MR1 (**Figure 12K**), and TCF12 (**Figure 12N**) were evenly distributed in immune cells and malignant cells. These results suggested that these immune genes may be the downstream regulators of m^6^A regulators and related lncRNAs participated in TME remodeling.

## Discussion

The TME plays a key role in different stages of tumorigenesis. Eyes are an immune-privileged site but inflammation can develop in an ocular tumor TME (24). UM is homogeneous without much stromal tissue, and therefore, it may be affected by immune cells (25). Compared with other malignancies, the presence of infiltrating macrophages and T cells in UM is associated with a poorer rather than a better prognosis (25), which was consistent with our findings. Moreover, previous studies have suggested that tumor-infiltrating macrophages and T cells are independent predictors for the prognosis of patients with UM (26, 27). In this study, results of the transcriptome analysis of UM data indicated that UM patients with high ImmuneScore had a poor prognosis. Besides, ImmuneScore was found to be significantly associated with many TICs such as T cells and macrophages. These results suggested that the TME played an important role in UM. Clarifying the mechanisms of the TME will provide novel insight into the development of highly effective immunotherapeutic strategies.

Post-transcriptional regulation is important for regulating the gene expression processes, which determine cellular function. Decades of research have identified more than 100 types of ribonucleosides that are post-transcriptionally modified (28). m^6^A methylation is one of the most prevalent post-transcriptional modifications found in eukaryotic mRNAs and lncRNAs (28, 29). More studies have reported that m^6^A regulators extensively participate in diverse biological processes and prognoses in different cancers (13, 14, 30, 31). A recent study has suggested that METTL3-mediated m^6^A methylation modulates UM cell proliferation, migration, and invasion by targeting c-Met (32). As far as we know, the role of m^6^A methylation in UM has less been studied, and the effect of m^6^A methylation on the TME of UM has not been fully understood.

In this study, we found that m^6^A regulators were related to the prognosis and TME of patients with UM. We established a prognostic risk signature using 3 m^6^A regulators based on OS. The signature helped differentiate UM patients into high- and low-risk groups and could serve as an independent risk factor for UM prognosis. The high-risk group was positively correlated with immune cell infiltration levels. Among the 3 m^6^A regulators, IGF2BP2 acts as m^6^A readers to enhance mRNA stability and translation and plays an important role in tumors (33). YTHDF3 functions as oncogenes in breast cancer (34). A recent study has investigated that ocular melanoma samples show decreased m^6^A levels, indicating a poor prognosis (35). In our study, patients with high RBM15B expression had a good prognosis. RBM15B acts as a methyltransferase and thus promotes the level of m^6^A RNA methylation. Therefore, it is reasonable to speculate that high levels of m^6^A methylation are beneficial to patients’ survival, which is consistent with the current study. These findings indicated that m^6^A regulators played an important role in the development and progression of cancer. However, the underlying mechanisms of m^6^A in tumor development still need to be further clarified.

**Figure 13:**
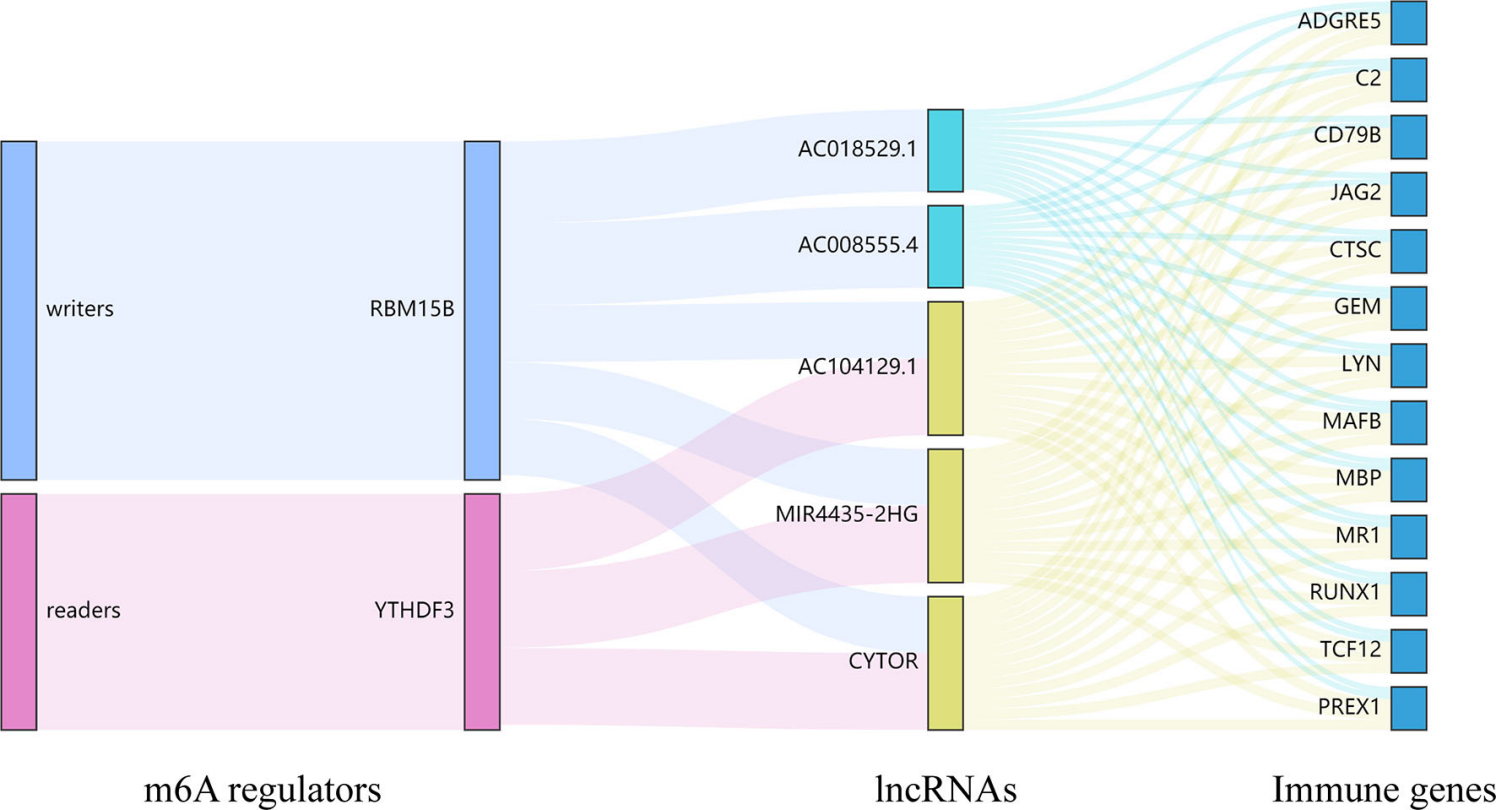
The Sankey diagram depicted the relationship between 2 m^6^A regulators, 5 lncRNAs, and 13 immune genes. m^6^A readers: YTHDF3; m^6^A writers: RBM15B; lncRNAs: AC008555.4, AC018529.1, AC104129.1, CYTOR, and MIR4435-2HG; immune genes: ADGRE5, C2, CD79B, CTSC, GEM, JAG2, LYN, MAFB, MBP, MR1, PREX1, RUNX1, and TCF12.

Currently, some studies have clarified the role of lncRNAs in UM. Among these lncRNAs, lncRNA PVT1 and R2RX7-V3 function as novel oncogenes and promote tumorigenesis (36, 37), whereas lncRNAs CANT1 and PAUPAR suppress tumorigenesis in malignant UM (38, 39). However, no study has analyzed the effect of m^6^A regulators-related lncRNAs on the TME and prognosis in UM. Here we identified m^6^A regulators-related lncRNAs by performing the correlation analysis and further screened lncRNAs based on OS and PFS. The cluster 1/2 subtypes identified through consensus clustering based on the expression of m^6^A regulators-related lncRNAs were also related to ImmuneScore, the prognosis of patients, and immune cell infiltration levels. Finally, 5 m^6^A regulators-related lncRNAs were found to be associated with the OS of UM patients. Among the 5 lncRNAs, lncRNA MIR4435-2HG targets desmoplakin and promotes growth and metastasis of gastric cancer by activating Wnt/β-catenin signaling (40). LncRNA-CYTOR and Wnt/β-Catenin signaling form a positive feed-forward loop to promote the metastasis of colon cancer (41). Through the correlation analysis, we screened 13 downstream immune genes targeted by 5 lncRNAs. They were all found to be upregulated in the high-risk group and cluster 2. At present, RUNX1, MR1, and PREX1 have been reported to be associated with T cells (42–44). CD79B is an important driver of immune-privileged site-associated diffuse large B cell lymphoma (45). JAG2 has been found to be overexpressed in malignant plasma cells from multiple myeloma patients and cell lines (46). These results suggested that lncRNAs may affect the immune cell infiltration through 13 common immune genes. Finally, we constructed a Sankey diagram that depicted the relationship between m^6^A methylation regulators, lncRNAs, and immune genes (**Figure 13**). These findings require further validation and may provide invaluable insights into the future treatment of patients with UM.

m^6^A methylation is a prevalent form of RNA modification that may provide a novel approach for tumor treatment. However, many key aspects, such as the regulatory mechanisms of m^6^A regulators and the unidentified relationship between m^6^A regulators and TME, remain to be explored. Therefore, in this study, we systematically explored the relationship between m^6^A regulators with the prognosis and TME in UM, further identified the potential lncRNAs and immune genes. However, further validation based on more clinical samples is required, and thus clinical samples will be collected to determine the level of m^6^A methylation and the association between the expression of m^6^A regulators and patients’ survival in the future. Furthermore, the downstream regulatory mechanisms of m^6^A regulators will be investigated to screen possible targets by methylated RNA immunoprecipitation sequencing (MeRIP-seq) and RNA-binding protein immunoprecipitation-quantitative polymerase chain reaction (RIP-qPCR). Tumorigenesis in animals and phenotypes of cell lines are necessary to explore the function of m^6^A regulators in this process.

In conclusion, our study provided an m^6^A regulators-based signature for prognostic prediction of UM and confirmed that m^6^A regulators and related lncRNAs played an important role in TME remodeling. These findings might provide promising targets for improving the survival of UM patients.

## Data Availability Statement

The datasets analysed for this study can be found in The Cancer Genome Atlas (https://portal.gdc.cancer.gov/).

## Author Contributions

ZL (1^st^ author) and ZL (5^th^ author) conceived the project. ZL (1^st^ author) performed the computational analyses and wrote the manuscript. SL designed and produced the figures. SH and TW contributed to the literature search for the manuscript. SL and ZL (5^th^ author) revised the manuscript. All authors contributed to the article and approved the submitted version.

## Conflict of Interest

The authors declare that the research was conducted in the absence of any commercial or financial relationships that could be construed as a potential conflict of interest.

## Publisher’s Note

All claims expressed in this article are solely those of the authors and do not necessarily represent those of their affiliated organizations, or those of the publisher, the editors and the reviewers. Any product that may be evaluated in this article, or claim that may be made by its manufacturer, is not guaranteed or endorsed by the publisher.

## Supplementary Material

Supplementary Figure 1 Identification of m^6^A regulators-related lncRNAs related to OS and PFS. **(A, B)** Forest plots for the univariate Cox analysis of prognosis based on OS **(A)** and PFS **(B)**. Colored dots represent hazard ratio, and the horizontal lines across the hazard ratio represent 95% confidence interval. OS, overall survival; PFS, progression free survival.
