# Supplementary figures and images for: N6-Methyladenosine Regulators and Related LncRNAs Are Potential to be Prognostic Markers for Uveal Melanoma and Indicators of Tumor Microenvironment Remodeling
Source: Front Oncol. 2021 Jul 30;11:704543. doi: 10.3389/fonc.2021.704543 (PMC8362329; doi:10.3389/fonc.2021.704543)

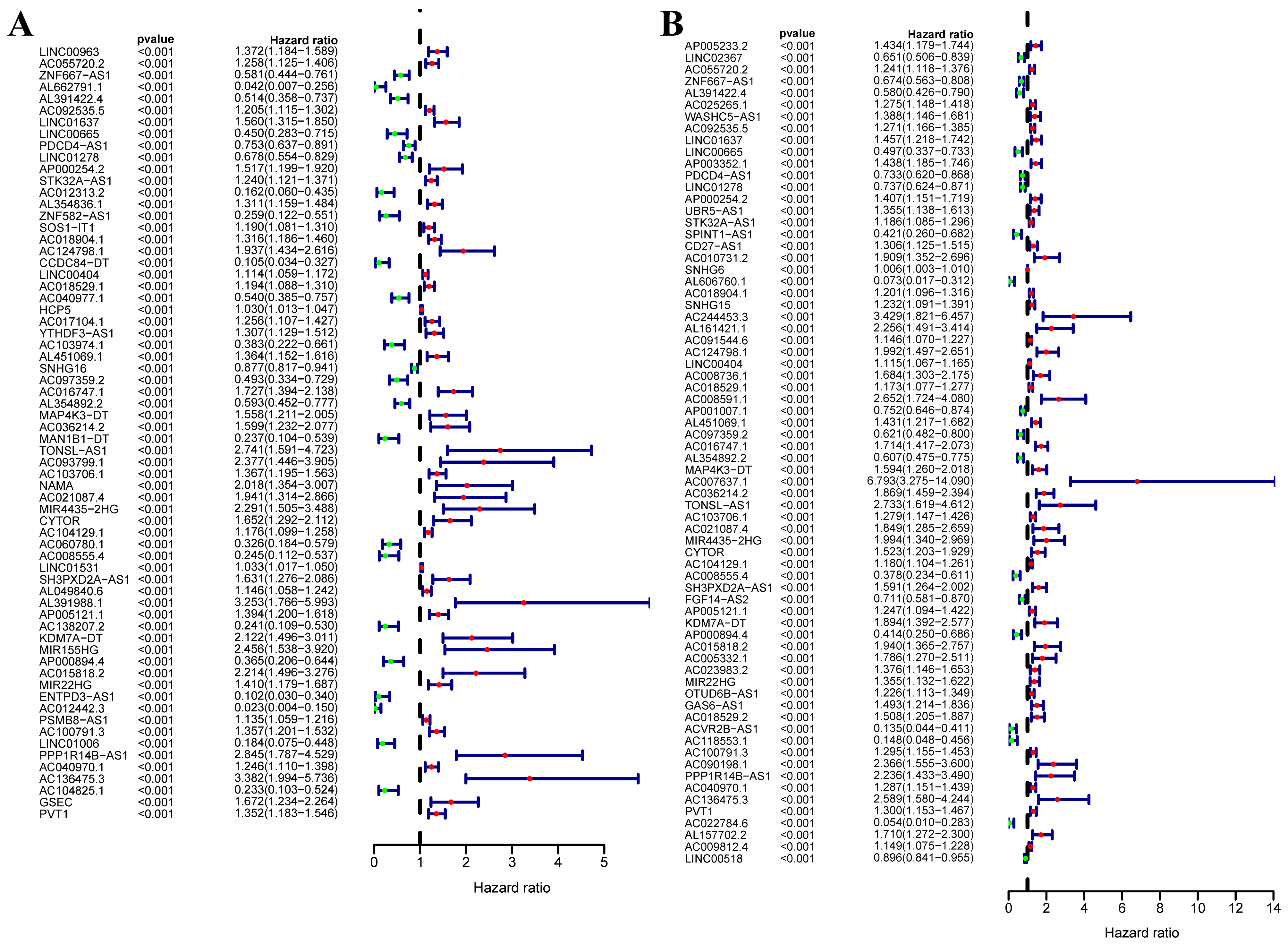

Supplement: Supplementary Figure 1 — Identification of m6A regulators-related lncRNAs related to OS and PFS. (A, B) Forest plots for the univariate Cox analysis of prognosis based on OS (A) and PFS (B). Colored dots represent hazard ratio, and the horizontal lines across the hazard ratio represent 95% confidence interval. OS, overall survival; PFS, progression free survival. [file Image_1.tif]
