# Supplementary material for: N6-Methyladenosine Regulators and Related LncRNAs Are Potential to be Prognostic Markers for Uveal Melanoma and Indicators of Tumor Microenvironment Remodeling
Source: Front Oncol. 2021 Jul 30;11:704543. doi: 10.3389/fonc.2021.704543 (PMC8362329; doi:10.3389/fonc.2021.704543)
Supplement: Supplementary file 2 [file Table_1.docx]

**Supplementary Table 1.** Clinical features of patients with UM in TCGA.

| Characteristics |  | Cohort (TCGA-UM, N=80) |
| --- | --- | --- |
| Age (years) | <=60 | 36 (45.00%) |
|  | >60 | 44 (55.00%) |
| Gender | Female | 35 (43.75%) |
|  | Male | 45 (56.25%) |
| T stage | T1 | 0 (0.00%) |
|  | T2 | 4 (5.00%) |
|  | T3 | 36 (45.00%) |
|  | T4 | 38 (47.50%) |
|  | Unknown | 2 (2.50%) |
| M stage | M0 | 73 (91.25%) |
|  | M1 | 3 (3.75%) |
|  | Unknown | 4 (5.00%) |
| N stage | N0 | 76 (95.00%) |
|  | N1 | 0 (0.00%) |
|  | Unknown | 4 (5.00%) |
| TMN stage | I | 0 (0.00%) |
|  | II | 36 (45.00%) |
|  | III | 40 (50.00%) |
|  | Ⅳ | 4 (5.00%) |
|  | Unknown | 0 (0.00%) |
